# Dynamics of the Apo µ-Opioid Receptor in Complex with Gi Protein

**DOI:** 10.3390/ijms241713430

**Published:** 2023-08-30

**Authors:** Mira Raya Paula de Lima, Rubem Francisco Silva Bezerra, David Denis Bento Serafim, Diniz Maciel Sena Junior

**Affiliations:** 1 Biological Chemistry Department, Universidade Regional do Cariri—URCA, Crato 63105-000, CE, Brazil; rubem.bezerra@urca.br (R.F.S.B.); david.bento@urca.br (D.D.B.S.); 2 Instituto Federal de Educação Ciência e Tecnologia do Ceará—IFCE, Juazeiro do Norte 63040-540, CE, Brazil

**Keywords:** G protein signaling, molecular dynamics, Guanosine Di-phosphate (GDP)

## Abstract

Opioid receptors, particularly the µ-opioid receptor (μOR), play a pivotal role in mediating the analgesic and addictive effects of opioid drugs. G protein signaling is an important pathway of μOR function, usually associated with painkilling effects. However, the molecular mechanisms underlying the interaction between the μOR and G protein remain poorly understood. In this study, we employed classical all-atom molecular dynamics simulations to investigate the structural changes occurring with the μOR-G protein complex under two different conditions: with the G protein in the apo form (open) and with the GDP bound G protein (closed, holo form). The receptor was in the apo form and active conformation in both cases, and the simulation time comprised 1µs for each system. In order to assess the effect of the G protein coupling on the receptor activation state, three parameters were monitored: the correlation of the distance between TM3 and TM6 and the RMSD of the NPxxYA motif; the universal activation index (A100); and the χ^2^ dihedral distribution of residue W293^6.48^. When complexed with the open G protein, receptor conformations with intermediate activation state prevailed throughout the molecular dynamics, whereas in the condition with the closed G protein, mostly inactive conformations of the receptor were observed. The major effect of the G protein in the receptor conformation comes from a steric hindrance involving an intracellular loop of the receptor and a β-sheet region of the G protein. This suggests that G-protein precoupling is essential for receptor activation, but this fact is not sufficient for complete receptor activation.

## 1. Introduction

The G Protein-Coupled Receptors (GPCRs) comprise a large group of structures located on the cell plasma membrane that participate in various intracellular signaling pathways, thereby regulating cellular functions such as metabolism, growth, and death [1]. They are the target of 30–50% of commercially available drugs [2]. GPCRs exhibit this versatility due to the variety of biochemical functions they can bind to small molecules, lipids, peptides, and nucleotides [3]. Structurally, they consist of seven (7) transmembrane helices (TMs), an extracellular amino domain (N-terminal), and an intracellular carboxy domain (C-terminal).

GPCRs can assume a variety of conformations, ranging from inactive to intermediate to active states [4]. Upon activation, GPCRs trigger their cognate G protein, which is a heterotrimeric protein comprising three subunits, namely G alpha (Gα), G beta (Gβ), and G gamma (Gγ) [5,6]. The G protein converts Guanosine Triphosphate (GTP) to Guanosine Diphosphate (GDP) [7]. Afterwards, the α monomer opens up to exchange GDP for GTP and then dissociates from the βγ dimer [8]. The G proteins are named Gαs (stimulatory), Gαi (inhibitory), Gα_q/11_ (phospholipase C activator), and Gα_12/13_ (GTPase activator), according to the similarities and function of their structures [9,10].

According to their sequence similarity, GPCRs are divided into six different classes, namely A, B, B1, C, D, and F [5]. Class A, also known as the rhodopsin-like class, encompasses the most significant number of receptors in humans, with approximately 700 receptors [6]. Among these are the opioid receptors, further divided into four subtypes: δ-opioid (δOR), κ-opioid (κOR), μ-opioid (μOR), and nociceptin/orphanin (NOP) receptor [8].

μOR has the Gαi as its cognate protein [11] and is mainly related to regulating physiological processes such as analgesia [12]. Both endogenous opioids, such as enkephalin and endorphin, and exogenous ones, such as morphine, target this receptor, efficiently triggering antinociceptive effects via the G protein signaling pathway [13].

The μOR experimental structure was first published in 2012 (PDB id 4DKL), with the receptor in the inactive conformation [12]. A few years later, the active conformation structure was also resolved (PDB id 5C1M) [14]. The crystallization of the μOR in its active form and coupled to the G protein (PDB id 6DDF) represents a significant milestone in understanding the receptor’s activation features [11]. This structure includes the murine µOR (from *Mus musculus*, like the previous ones), the human heterotrimeric G protein (from *Homo sapiens*), and the peptide ligand DAMGO (D-Ala(2)-mephe(4)-gly-ol(5)) [11]. Since the murine receptor shares a high (94%) sequence and binding site identity with the human protein, this structure provides valuable insights that can be extended to its human counterpart [12]. Other µOR-Gi systems were crystallized with ligands like morphine and fentanyl (PDB id: 7SBF, 7SCG, 7U2K, 8EF6) [15,16,17]. All these complexes lack GDP or GTP and comprise the G protein in the open form. The heterotrimeric G protein with GDP is found in five structures (PDB id: 1GP2, 1GG2, 5TDH, 6CRK, and 7TD0) [18,19,20,21]. All of these comprise the inhibitory G protein in its closed form with GDP, serving as reference to build a model with the µ-opioid receptor. The structure with PDB id 1GP2, however, is the one that better superimposes the G protein in the PDB id 6DDF structure.

Detailed investigation of the GPCR structures and their interactions with new drugs can be a challenging and costly process due to the high expenses associated with the equipment and materials used for isolating and crystallizing reference structures [22]. To address these challenges, molecular modeling studies have become essential research tools [23]. These techniques include ab initio calculations, homology modeling, molecular docking, and molecular dynamics (MD) simulations [24]. In MD simulations, the behavior of molecular systems is observed over time using classical mechanics equations to predict the interactions, affinity, and compound stability under specific conditions like temperature and the presence of solvents, lipid membranes, or other cofactors [25]. With improved resolution of the crystal structures, computational power, and algorithm developments, molecular dynamics results can achieve a high degree of agreement with the experimental data [26]. Sena et al. successfully captured an active-like conformation of the μOR through enhanced sampling techniques, starting with an inactive conformation of a constitutively active mutant of the receptor [27]. This active-like conformation closely resembled the crystal structure released with PDB id 5C1M. Mafi et al. employed metadynamics and free energy calculations to study the human μOR structure in complex with Gi [28], where important interactions between the receptor and the G protein have been described.

In this paper, we present 1 μs long classical molecular dynamics simulations results employing two different complexes of the murine μOR (in active form) with the human inhibitory G protein. In one of the complexes, the G protein is in an open state, without any ligand, while in the other, the GDP bound G protein in a closed state is used. We are particularly interested in observing: (a) whether the coupling to the G protein is capable of stabilizing the receptor in its active-like state along the simulation; (b) changes in receptor’s structure and their differences based on the G protein state the receptor is complexed to; (c) the influence of the receptor in the opening or closing of the G protein, as well as the possibility of a detachment of the βγ subunits from the α subunit.

## 2. Results and Discussion

GPCRs, as membrane proteins, are susceptible to the presence of lipid molecules in the membrane, which can influence the receptor’s activation state and favor interaction with agonists [29]. Our models employed POPC, which is among the most common lipids in mammals [30]. In both systems, the receptor is similarly inserted in the membrane, such that possible allosteric effects can be equally leveled (see Supplementary Materials, Supplementary Figures S1 and S2).

**Figure 1:**
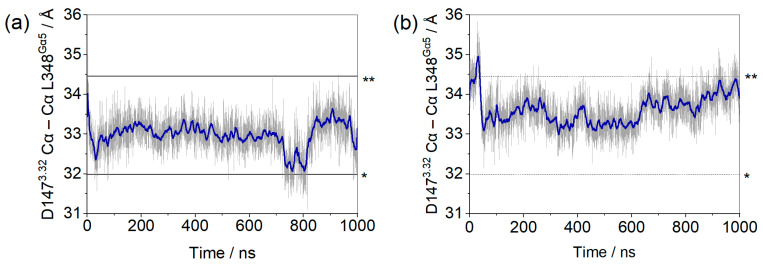
Distances between the Cα of residue D147^3.32^ of the µOR binding site and residue L348^Ga5^ of the Gα monomer for the µOR-Gi apo (**a**) and µOR-Gi-GDP system (**b**). The blue line represents the adjacent averaging of 50 points during 1000 ns. The dashed lines mark the minimum (*) and maximum (**) values measured on the available structures (listed in Supplementary Table S1).

After 1 µs of classical molecular dynamics simulations, the insertion of the α5 helix of the G protein, as measured by the distances between the alpha carbons of residues D147^3.32^ and L348^Gα5^, remained within a 4 Å interval, as shown in Figure 1. We proposed to monitor these residues due to their significance (D147^3.32^ is a conserved residue comprising the receptor´s binding pocket) and stability (L348^Gα5^ is close to but more stable than F354^Gα5^). Comparison with the equivalent distances measured on recently published structures of µOR-Gi complexes shows that, despite some variations, the observed values were within expectations (Figure 1 and Supplementary Table S1) [11,15,16,17,31,32].

**Figure 2:**
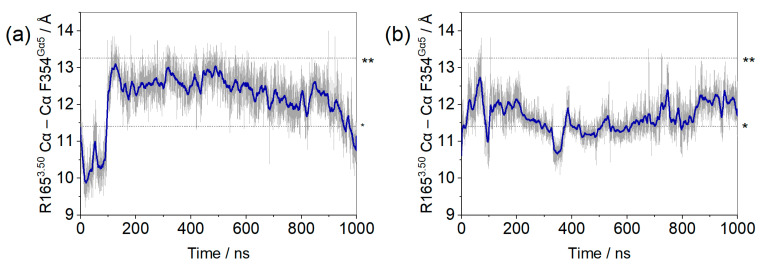
Distances between the Cα of residue R165^3.50^ of the µOR and residue F354^Gα5^ of the Gα monomer for the µOR-Gi apo (**a**) and µOR-Gi-GDP system (**b**). The blue line represents the adjacent averaging of 50 points during 1000 ns. The dashed lines mark the minimum (*) and maximum (**) values measured on the available structures (listed in Supplementary Table S1).

**Figure 3:**
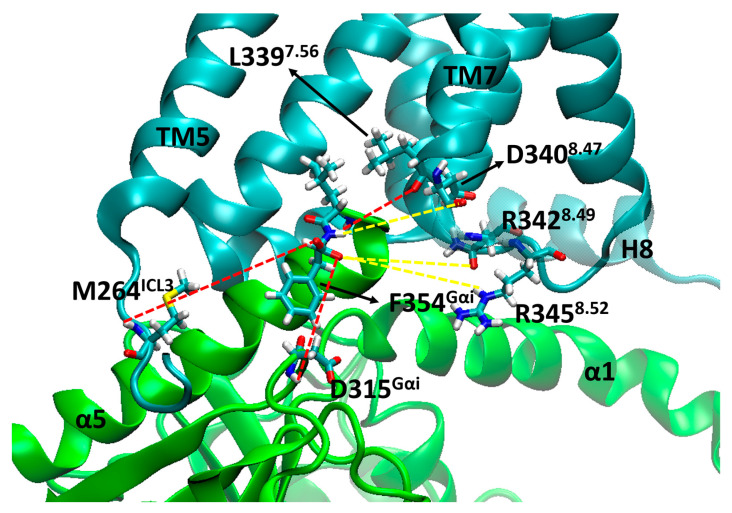
Contacts observed during MD for F354^Gαi^ on the µOR-Gi complex, where the receptor is cyan and the Gαi is green. The red dashed lines represent lost contacts and yellow dashed lines represent new contacts. TM6 was omitted, and H8 is transparent for the better visualization of residues.

Within the first 100 ns of simulation, it can be seen that residue F354^Gα5^ (at the end of the α5 helix) changed position in the µOR-Gi apo, increasing the distance between the receptor and the G protein, while the COM of the receptor and the Gαi protein maintained the same distance along the entire trajectory (see Supplementary Figure S3), as shown in Figure 2. The accommodation of F354^Gα5^ can be understood when its contacts are analyzed: in the first 100 ns, among others, the contact residues were M264^ICL3^, L339^7.56^, D315^Gα^; then, these interactions were broken and new ones were formed with M281^6.36^, D340^8.47^, N342^8.49^, and R345^8.52^ (Figure 3). In the last 200 ns of the dynamics, the interaction between F354^Gα5^ and M264^ICL3^ was re-established, reducing the distance between R165^3.50^ and F354^Gα5^ (Figure 2). These large variations can be understood considering that the F354^Gα5^ residue is at the end of the α5 helix and therefore has high mobility. Our observations are in agreement with those of Mafi et al. in their study of the µOR and Gi model, where they observed interactions between R165^3.50^ e F354^Gα5^ during their entire trajectories [25]. For the µOR-Gi-GDP system, this parameter showed a smaller average value but still oscillated within the expected limits.

**Figure 4:**
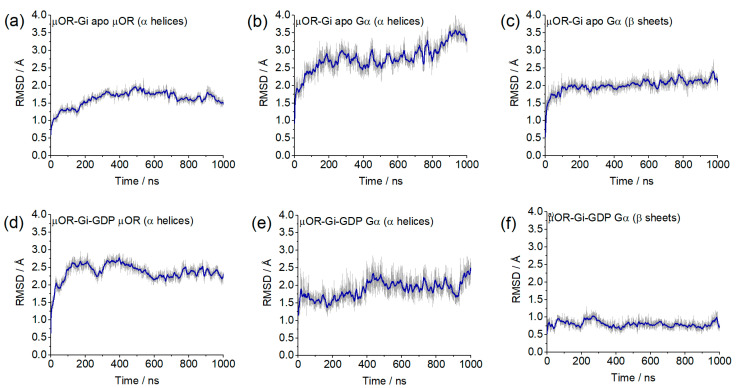
RMSD values from the Cα of the µOR and the Gα protein for selected portions/systems: (**a**) α-helices µOR/µOR-Gi apo; (**b**) α-helices Gα/µOR-Gi apo; (**c**) β-sheets Gα/µOR-Gi apo; (**d**) α-helices µOR/µOR-Gi-GDP; (**e**) α-helices Gα/µOR-Gi-GDP; (**f**) β-sheets Gα/µOR-Gi-GDP.

The stability of the system was evidenced by the RMSD values of each individual protein (µOR, Gα, Gβ and Gγ) using the backbone of the α-helices or β-sheets. In Figure 4, it can be observed that most of the values were between 0.5 and 2.5 Å, indicating maintenance of the system positions concerning the initial structure of the dynamics. The only portion that showed increasing RMSD and values greater than 2.5 Å were the α-helices of Gα (Figure 4b) in the µOR-Gi apo system, and this variation is related to the absence of the GDP and the open form of the protein but did not indicate a severe displacement of this portion. The maintenance of the Gα position can be inferred by the RMSD values of its β-sheet region (Figure 4f). The RMSD values from Gβ (β-sheets) and Gγ (α-helices) show that these proteins remained stable during the simulation, except for the Gγ in the µOR-Gi-GDP system, as shown in Supplementary Figure S4.

The effect of the G protein coupling to the receptor´s activation state was assessed by considering three parameters: the correlation plot between the TM3-TM6 distance and the RMSD of the NPxxYA motif [33], the Universal Activation Index (A^100^) [34], and the distribution of the χ^2^ dihedral angle values of residue W293^6.48^ [35].

**Figure 5:**
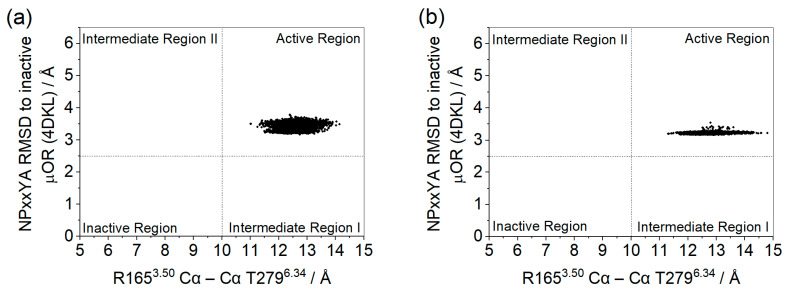
Distances between the Cα of residues R165^3.50^ and T279^6.34^ and the RMSD of the NPxxYA motif relative to the inactive µOR (PDB id 4DKL) (Å) for µOR-Gi apo (**a**) and µOR-Gi-GDP (**b**).

In Figure 5, it can be seen that, according to the first criterion, for both systems, the receptor remained in an active conformation. However, in the system with GDP there was a wider range in the TM3–TM6 distance, whereas in the apo system the NPxxYA RMSD shows a larger variation.

**Figure 6:**
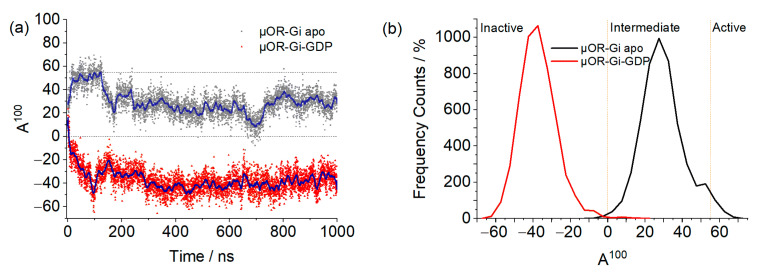
Graph (**a**) shows the A^100^ values for the µOR-Gi apo (black) and µOR-Gi-GDP (red) systems. The blue lines represent the moving averages of 50 points, while the orange ones represent the activation state classification threshold (A^100^ < 0 is inactive, 0 < A^100^ < 55 is intermediate, and A^100^ > 55 is active). Graph (**b**) shows the Frequency Count (%) of the A^100^ values obtained for both systems.

**Figure 7:**
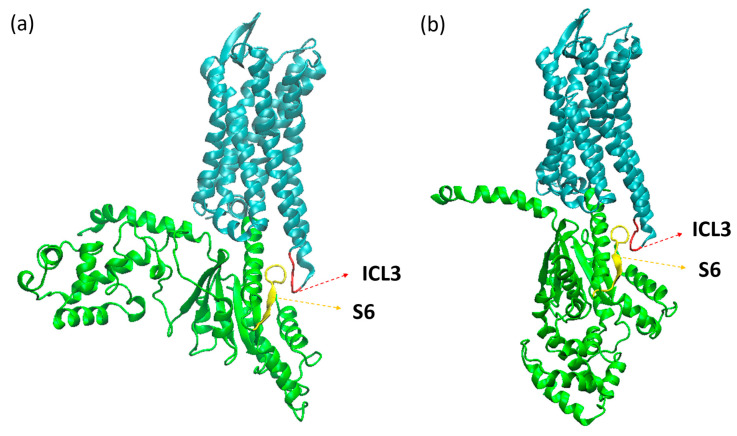
Structures of the µOR-Gi apo (**a**) and µOR-Gi-GDP (**b**) systems with highlighted portions, ICL3 (red), S6 (yellow), receptor (cyan), and Gα monomer (green).

With the A^100^, we can observe distinct behaviors between systems, as shown in Figure 6. Both systems started the simulation with the receptor in an intermediate state of activation, as predicted by the A^100^ index. For the apo system, it can be seen that in the beginning of the simulation, the receptor was found in the active state, then changed conformation to the intermediate state, and so remained for the rest of the trajectory, with some sporadic frames reaching the inactive state. The system with GDP, in its turn, soon changed to the inactive state and remained as such for the rest of the trajectory. This difference between the systems may be related to the geometry of the G protein: when open, it prevents the movement of the helices in the intracellular part of the receptor, preventing it from assuming an inactive conformation. In µOR-Gi-GDP, where the G protein is closed, there is no steric hindrance to the movement of the TMs in the intracellular portion of the receptor, which contributes to a change from the intermediate state to the inactive state. The closed form of the G protein is achieved after the Ras-like domain approaches the AH domain in order to accommodate a GTP molecule (later on turned into GDP). This movement is responsible for the β-sheet S6 moving away from the receptor, giving room to the ICL3 and allowing the receptor to change conformation. In Figure 7, the ICL3 and S6 portions can be observed.

**Figure 8:**
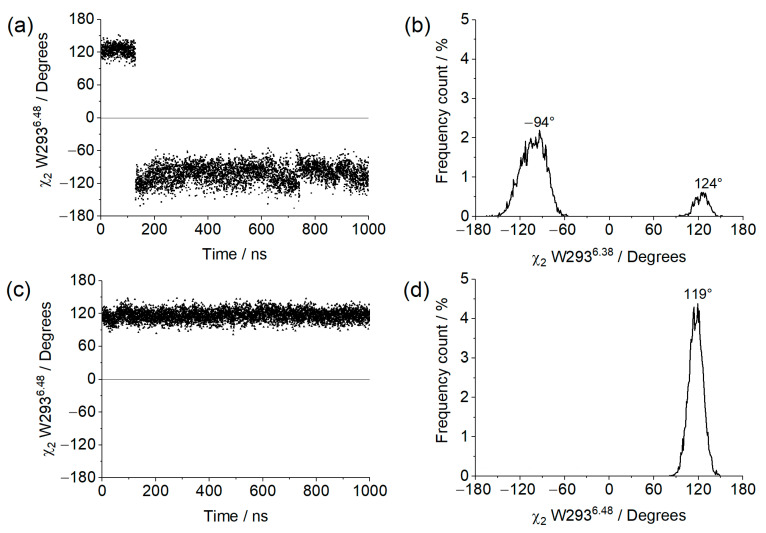
χ_2_ of W293^6.48^ values obtained from the 1µs MD simulation for (**a**) µOR-Gi apo, (**c**) µOR-Gi-GDP, and the respective frequency counts (%) (**b**) and (**d**), respectively.

The χ_2_ dihedral angle of residue W293^6.48^ along the trajectory, as well as its frequency distribution, is shown in Figure 8 for both systems. In this regard, the apo system behaved similarly to what was previously observed in simulations of the µOR bound to BU72, where one of the clusters with high values of NPxxYA RMSD and TM3–TM6 distance presented a practically identical distribution [36]. The system with GDP presented an angle distribution closer to the one presented by the two other clusters of µOR-BU72 complex but with no negative values at all. It should be noted that the receptor structure in these simulations was derived from PDB id 5C1M; so, it can be inferred that the presence of the ligand is responsible for driving the W293^6.48^ angle towards negative values, and this effect was also observed for the apo receptor in complex with the open Gαi. This is consistent with the fact that when coupled to the open Gαi, the receptor is more likely to assume an intermediate/active conformation.

**Figure 9:**
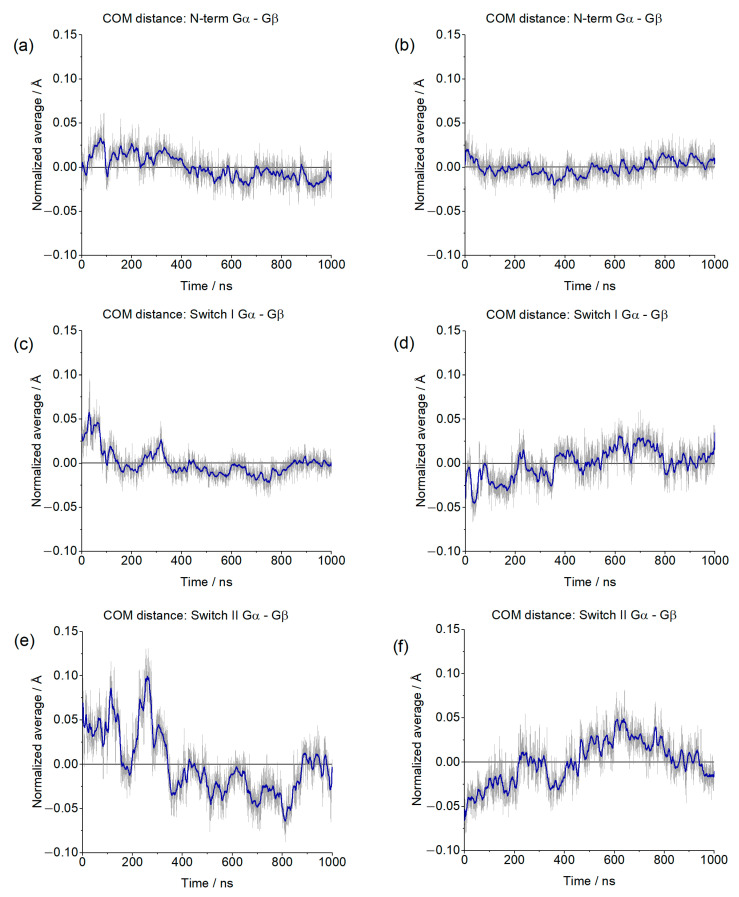
Normalized distances between the Center of Mass (COM) of the Gα N-terminal (**a**,**b**), Switch I (**c**,**d**), and Switch II (**e**,**f**) and the COM of Gβ. The blue line corresponds to the adjacent averaging of 50 points.

The overall stability of the G protein ternary complex was assessed by evaluating the distances of different regions of Gαi (N-terminal, switch I, and switch II) from the Gα. The centers of masses were considered, and due to the dissimilarity between our systems, deviations from the average values (normalized) were used. These results are shown in Figure 9.

**Figure 10:**
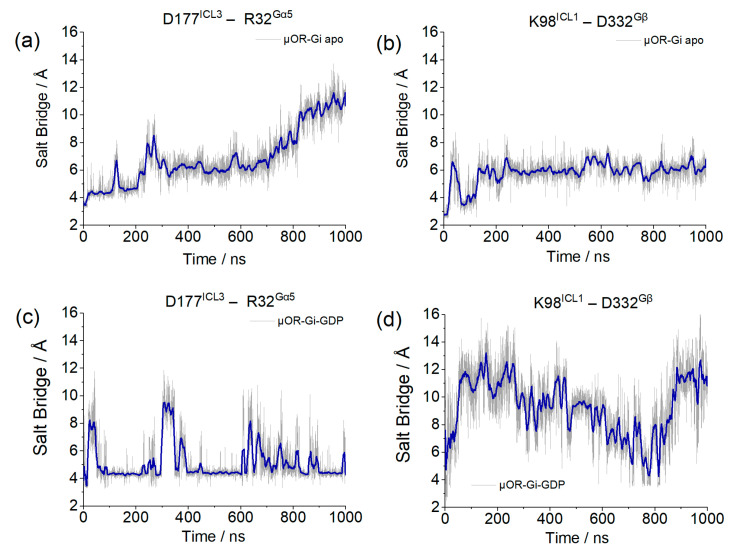
Salt bridge distances between D177^ICL3^ and R32^Gαi^ and between K98^ICL1^ and D332^Gβ^ for system µOR-Gi apo (**a**,**b**) and µOR-Gi-GDP (**c**,**d**). The blue line corresponds to the adjacent averaging of 50 points.

The parameters for the verification of the coupling between the receptor and G protein are salt bridges formed between the receptor and the Gα monomer, through residues D177^ICL3^ and R32^Gαi^, and between the receptor and Gβ, through residues K98^ICL1^ and D332^Gβ^. The lengths of these salt bridges are shown in Figure 10.

Although the salt bridge distance involving the receptor and Gα increased in the µOR-Gi apo system, the one involving Gβ remained stable, and this movement did not correlate with the Gα insertion discussed before, meaning that the G protein was not moving away from the receptor within the simulation time. An opposite behavior was presented by the system with GDP, where the salt bridge between the receptor and Gα persisted, despite some variations, and the one of the receptors with Gβ was broken.

**Figure 11:**
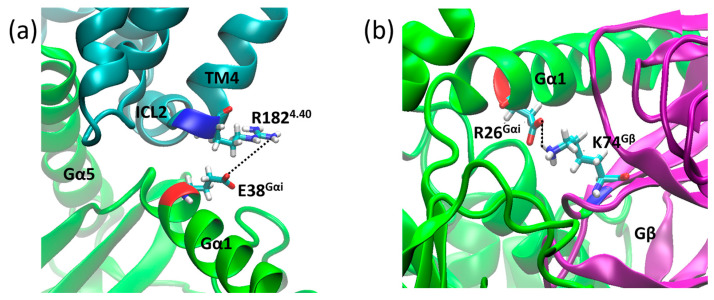
Salt bridge distances in the µOR-Gi apo between residues R182^4.40^ (blue) and E38^Gαi^ (red) (**a**) and D26^Gαi^ (red) and K74^Gβ^ (blue) (**b**).

**Figure 12:**
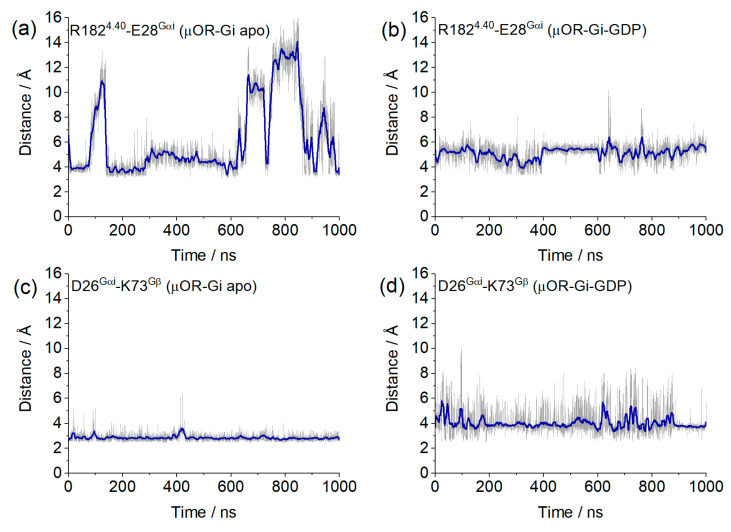
Values of the salt bridge distances obtained from the µOR-Gi apo and µOR-Gi-GDP between residues R182^4.40^ and E28^Gαi^ (**a**,**b**) and D26^Gαi^ and K73^Gβ^ (**c**,**d**). Blue lines correspond to the adjacent averaging of 50 points.

In addition to the bridges monitored above, we found two salt bridges common to both systems. The first salt bridge found was between R182^4.40^ and E28^Gαi^ (Figure 11a), and the second was between D26^Gαi^ and K74^Gβ^ (Figure 11b), which contribute to receptor G protein coupling and to Gαi and Gβ coupling, where the distance values of these bridges are shown in Figure 12. Other salt bridges found in each system are listed in Table S2 in the Supplementary Material.

**Figure 13:**
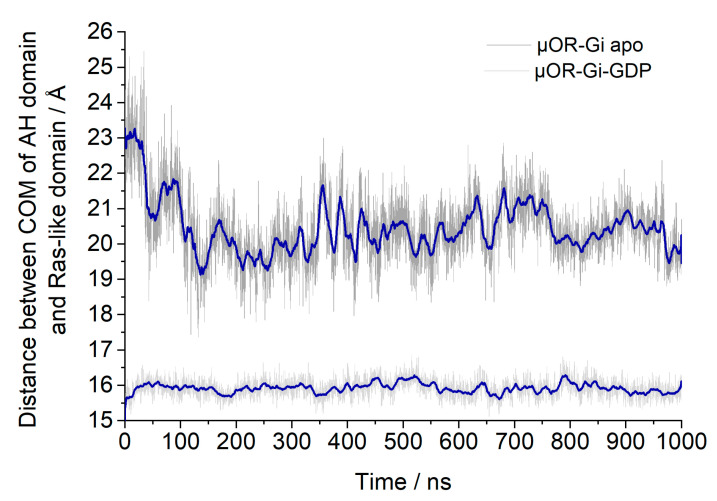
Distances between the Centers of Mass (COM) of the AH domain and the Ras-like domain to assess the possible GDP binding site opening. The blue line shows the adjacent averaging of 50 points.

Another structural aspect evaluated was the opening of the GDP binding site, which was monitored through the distances between the centers of mass (COM) of the AH (alpha helical) domain and the Ras-like domain of the Gα monomer [28]. These are shown in Figure 13.

For the apo system the distance between the AH and Ras-like domains was reduced in the beginning of the simulation by approximately 3 Å; then, it remained stable, as expected in order to allow the GTP to enter again. For the other system, it can be inferred that the presence of GDP stabilized the Gα portion in the closed state, and the expected opening triggered by the receptor activation might be achieved only after an agonist is bound to the receptor or maybe after a longer simulation time.

## 3. Materials and Methods

Two models of the receptor-G protein complex were constructed using the receptor’s structure in the active state and without ligand, as obtained from GPCRdb (refined from PDB id 5C1M) [37]. The experimental G protein structure in the available complex (PDB id 6DDF) was not completely resolved; so, these structures were kindly supplied by Prof. Goddard III’s group, who have modeled the Gi apo structure based on the PDB id 6DDF [16] and the Gi-GDP structure based on PDB id 1GP2 [21]. G protein structures with GDP and without the MOR receptor are also available in PDB, such as PDB id 6CRK and 1GP2 (see Supplementary Table S3) [20,21]. Despite the slightly lower resolution (2.3 Å versus 2.0 Å), the latter was chosen to model the system due to a better alignment within the receptor/G protein interface. In the first model, the µOR is bound to an open-form G-protein without GDP (µOR-Gi apo), while in the second model it is bound to a closed-form GDP bound G-protein (µOR-Gi-GDP).

Among the structures available in the PDB, complexes with µOR-Gi without the GDP (listed in Supplementary Table S4 in the Supplementary Material), comprise the µOR receptors from *Homo sapiens* and *Mus musculus* species. The similarity between these receptors is high (94%), and the resolution of the active conformation in the 5C1M crystal allows us an approximate reference to reality for model construction. The G proteins in these structures originate from species such as *Rattus novergicus*, *Homo sapiens*, and *Bos taurus* [11,15,16,17,31,32]. These G proteins also show a high degree of similarity, greater than 85%.

### 3.1. µOR—G-Protein Complex without GDP

The µOR-Gi apo complex was constructed by aligning the receptor and G protein structures with those from PDB id 6DDF. The interface between the receptor and Gα was maintained by aligning the Gα1 (residues L5^α1^ to E30^α1^) and Gα5 (residues T340^Gα5^ to F354^Gα5^) using the UCSF Chimera program and taking the corresponding alpha carbons (Cα) as reference [38].

### 3.2. µOR—G-Protein Complex with GDP

We remodeled the Gα1 (L5^Gα1^ to A31^Gα5^) and Gα5 (T329^Gα5^ to F354^Gα5^) portions of the G protein using the SwissModel server [39] using the corresponding residues from PDB id 6DDF. Then, the complex was aligned to the structure in PDB id 6DDF as a template, in order to fix any clashes with the lipid membrane. This adjustment favored a more objective analysis of the µOR-Gi behavior with and without GDP. The initial pose of GDP was set after alignment of our model with the structure of PDB id 1GP2, using the alpha helical (AH) and Ras-like domains as references [21].

### 3.3. GDP Parametrization

The initial GDP structure was taken from PDB id 1GP2 [21] and optimized using G.A.M.E.S.S. 2018 R3 [40] with an HF level of theory and 6–31G(d,p) basis set. The charge parameters were extracted using RESP 2.3 [41] and parsed with Antechamber from Amber 21 [42]. The ligand topology was built with the General Amber Force Field (GAFF) v 1.81 [43] and converted to gromacs format using ACPYPE 2021.2.5 [44].

### 3.4. Membrane Building

The membrane was generated with 240 molecules of 1-palmitoyl-2oleoyl-sn-glycero-3-phosphocholine (POPC) per leaflet using the MemGen server [45]. The initial structure of the POPC molecule and Area per Lipid (APL) parameter was taken from Stockholm Lipids [46]. The APL was set to 63 Å^2^/POPC, and 80 molecules of water were added per POPC molecule on each side of the membrane [46]. The membrane–water ensemble energy was minimized at first with the steepest descent algorithm [47] followed by the conjugate gradient algorithm with flexible water molecules, with no position restraints. Afterwards, the system was subjected to NVT equilibration, followed by NPT equilibration, for 500 ps long each, using a Beredsen barostat [48] and V-rescale thermostat [49]. Finally, a production run was performed for 2500 ps in order to monitor the membrane stability. All MD steps used gromacs 2021.3 software.

### 3.5. Structures Topologies

The topologies of the structures were generated to be compatible with the gromacs 2021.3 program. The selected force field was amber99sb-ildn [50], adapted to include the parameters for the GDP and POPC (from Stockholm Lipids) [46].

### 3.6. Membrane Insertion and Solvation

**Figure 14:**
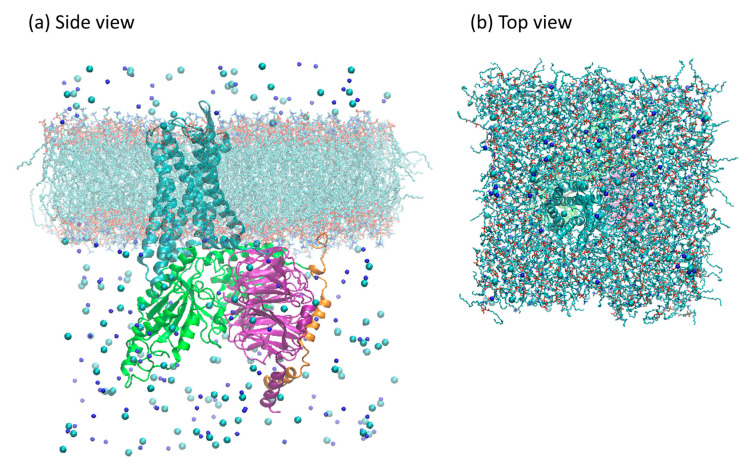
Model of the ligand-free µOR-Gi complex used in the simulations. Proteins are shown as ribbons: µOR in cyan, Gαi in green, Gβ in purple, and Gγ in orange. Ions are shown as spheres: Na^+^ in dark blue, and Cl^−^ in cyan. Lipids are shown as sticks; hydrogens not shown. Water molecules are omitted for clarity. In panel (**a**), the view from the side, and in panel (**b**), the view from the top.

The receptor was aligned with the z-axis according to the Orientations of Proteins in Membranes (OPM) database [51], then embedded into the POPC membrane using the embed tool available in gromacs 2018 [52]. After this procedure, 19 POPC molecules were removed from each leaflet, resulting in a final membrane patch with 221 POPC molecules per leaflet. The Gi protein counterpart was aligned to the embedded receptor according to the complexes models previously described. The final adduct was solvated using the TIP3P model for water [53]. Water molecules, which were eventually added within or between the upper and lower leaflets, were manually removed. NaCl was added for complete charge neutralization, up to a 150 mM concentration, to better reproduce physiological conditions [54]. A depiction of the simulation system can be seen in Figure 14. The system dimensions, in terms of the particles, are listed in Supplementary Table S5.

### 3.7. Energy Minimization

The structures were subjected to energy minimization that occurred in 3 steps. The first step consisted of energy minimization with the steepest descent algorithm [47] with position restraint for protein, lipids, and GDP (when present). In the second step, position restraints were set for the proteins only. The last minimization step was performed without any restraints using the conjugate gradient algorithm with flexible water molecules. All minimization steps considered an upper total energy threshold of 100 kJ/mol/nm. The systems were subsequently subjected to NVT equilibration, followed by NPT equilibration, each one 500 ps long. The temperature was controlled at 300 K by coupling to the V-rescale thermostat [55], and the pressure was set to 1 bar by coupling to the C-rescale barostat [49].

### 3.8. Molecular Dynamics

Molecular dynamics runs for both systems were carried out using gromacs 2021.3 [52], with a reference temperature of 300 K and 1 bar of pressure, with a timestep of 2 fs. The thermostat used for the equilibration and the MD production steps was the modified Berendsen thermostat using velocity rescaling (V-rescale), and the barostat was the C-rescale, where the relaxation pressure is coupled to the time constant and with correct volume fluctuations [49].

### 3.9. Assessment of Complex Contacts

**Figure 15:**
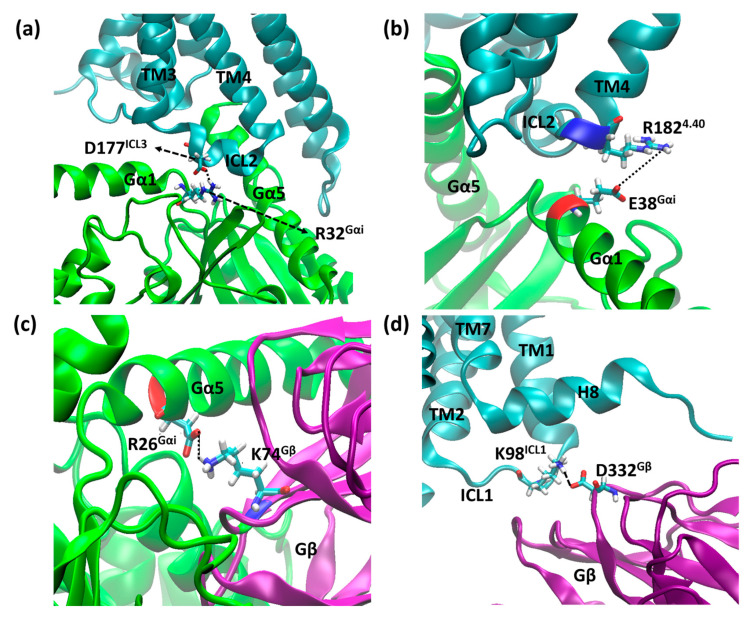
Residues D177^ICL3^–R32^Gαi^ (**a**), R182^4.40^–E28^Gαi^ (**b**), D26^Gαi^–K73^Gβ^ (**c**), and K98^ICL1^–D332^Gβ^ (**d**), which may form salt bridges between the receptor and Gα and the receptor and Gβ, where µOR is in cyan, Gαi is in green, Gβ is purple, and the residues’ side chains are shown as sticks.

As our simulations involve multiprotein systems, strong interactions were assessed along the trajectories. Salt bridges were located using the default parameters in VMD 1.9.4 [56]. Some of them, common to both structures, were selected: D177^ICL3^–R32^Gαi^ (Figure 15a), R182^4.40^–E28^Gαi^ (Figure 15b), and D26^Gαi^–K73^Gβ^ (Figure 15c). In addition to those, Mafi et al. reported a salt bridge between receptor and Gβ, namely K98^ICL1^–D332^Gβ^ (Figure 15d), which was also monitored.

**Figure 16:**
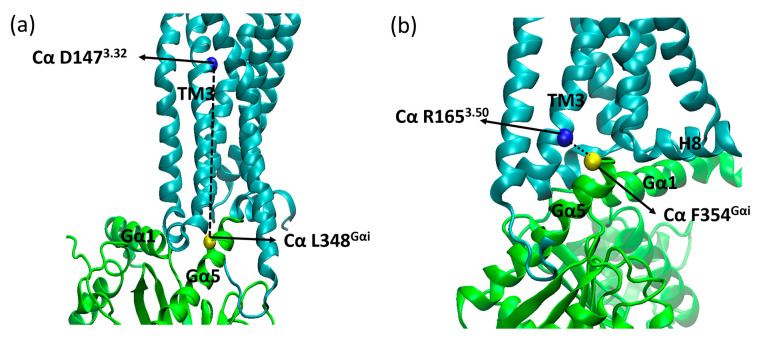
Parameters used to assess complex stability. Gα5 insertion into the receptor from the distance between Cα of D147^3.32^ (blue sphere) and Cα of L348^Gαi^ (yellow sphere) (**a**), Cα of R165^3.50^ (blue sphere) and Cα of F354^Gαi^ (yellow sphere) (**b**), where TM6 was omitted for better visualization, where µOR is in cyan, Gαi is in green, Gβ is purple, and the residues’ side chains are shown as sticks.

**Figure 17:**
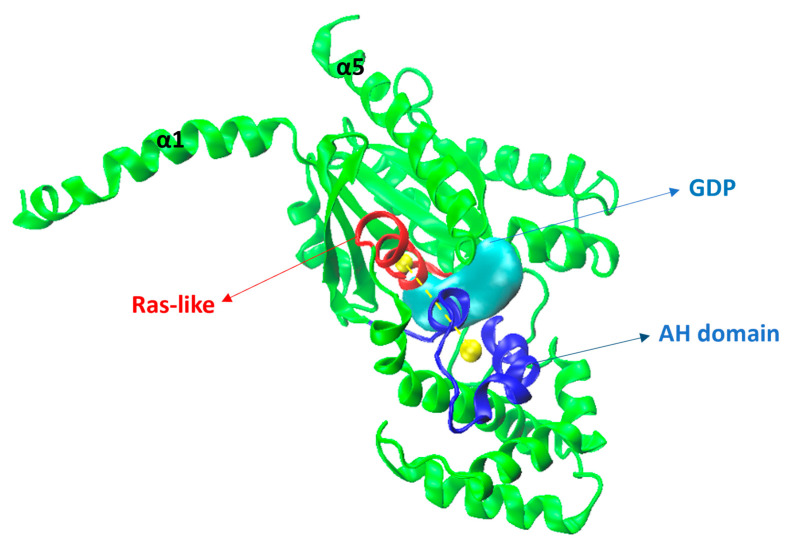
Gαi (green) model showing the Ras-like portion is in red, the alpha helical domain (AH) in blue, and the GDP (as surface, in cyan). Yellow spheres show the center of mass (COM) and the yellow dashed line indicates the distance between the COMs of the Ras-like and AH portions.

**Figure 18:**
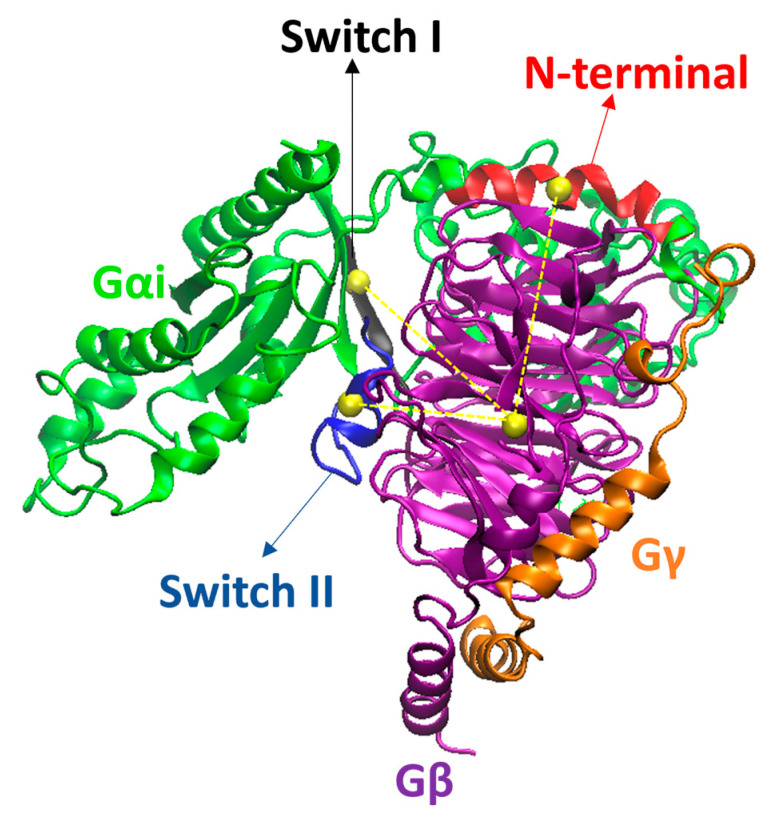
Gαi, Gβ, and Gγ from the µOR-Gi-GDP system, in green, purple, and orange, respectively. The N-terminal (N-term), Switch I (SwI), and Switch II (SwII) regions of Gαi are represented in red, black, and blue, respectively. The yellow spheres represent the centers of mass (COM) of the regions in which they are inserted, while the dashed yellow lines represent the distances between the COM of the areas in Gαi and the COM of Gβ. Residues R67^Gαi^, T265^Gαi^, and L355^Gβ^ were omitted for better visualization of the COM spheres.

Other parameters considered to assess the stability of the complexes were the insertion of the α5 helix of Gαi into the receptor (distances between Cα of D147^3.32^ and L348^Gα5^, Cα of R165^3.50^, and F354^Gα5^) (Figure 16) and the distance between the centers of mass (COM) of the AH and Ras-like portion of Gαi (Figure 17). The contacts between the G protein monomers were monitored through the distances between the COM of the entire Gβ monomer and the N-terminal region of Gα (residues A5^Gα1^ to G37^Gα1^), the Switch I region of Gα (residues R179^swI^ to F189^swI^), and the Switch II region of Gα (residues G203^swII^ to V218^swII^) (Figure 18) [9].

### 3.10. RMSD Calculations

RMSD values of the studied systems on the protein backbone were calculated. The α-helices were the target regions for the µOR and Gγ, while for the Gα, α-helices and β-sheets were considered. The β-sheets were the target regions for the Gβ. The position at 0 ps of the production dynamics was used as a reference. All trajectory frames had the backbones of target portions aligned to the reference frame for this calculation, and then the RMSD was calculated using VMD 1.9.4 software [56].

### 3.11. Assessment of Receptor Activation States

The degree of activation of the receptor along the simulation was assessed by measuring the Universal Activation Index (A^100^) [34] and by correlating the distance measured between the Cα of residues R165^3.50^ and T279^6.34^ with the RMSD of the NPxxYA motif (without hydrogens) using the receptor´s inactive crystal structure (PDB id: 4DKL) as reference [33]. The receptor is classified as inactive when the A^100^ values are negative, intermediate for the A^100^ values between 0 and 55, and active for A^100^ values greater than 55 [34]. The χ^2^ dihedral of the W293^6.48^ residue is also related to activation states of the receptor and was monitored along the simulations. The active conformations present negative dihedral values close to −92° and/or positive values close to 117° [9].

## 4. Conclusions

In this study, we prepared molecular models for two systems comprising the murine µOR and its cognate G protein. We observed that, when the receptor was coupled to the open G protein, intermediate/active conformations were stabilized. On the other hand, when the receptor was coupled to the closed form of the G protein, inactive conformations were favored. These differences can be directly attributed to the steric hindrance caused by the S6 portion of Gαi when in the open form, which prevents the movement of the ICL3 from the µOR and, consequently, the movement of TM6. When the G protein is closed, such steric hindrance is absent, allowing the receptor to change conformation towards inactive states.

The NPxxYA motif in the receptor, a well-known activation toggle switch, assumed different conformations, depending on whether the receptor was bound to the open or closed form of the G protein. In the former case, conformational changes of this motif were more pronounced, as evidenced by the larger RMSD variations than in the latter case.

We also observed that in the absence of an agonist, µOR coupling was not able to alter the closed form of the GDP bound G protein, nor was any sign of decoupling of the Gβγ observed. In fact, if the closed form of the G protein favors an inactive conformation of the receptor, one might expect that the presence of an agonist might be necessary to trigger any G protein changes. The open G protein, however, was able to stabilize the receptor in an intermediate state, from which it could be activated, also upon agonist binding. Additional studies will consider ligand-bound complexes to verify the combined effect with the G protein.

In addition, it was possible to identify throughout the research that recently published structures from systems similar to ours showed residue distances similar to those observed in our simulations. Therefore, these models will open the way for new studies involving the ligand-bound receptor-G protein complexes.

## Data Availability

The data presented in this study are openly available in Mendeley Data at https://dx.doi.org/10.17632/9dvbntsmtv.1.

## Supplementary Materials

The following supporting information can be downloaded at: https://www.mdpi.com/article/10.3390/ijms241713430/s1.
