# Supplementary material for: Dynamics of the Apo µ-Opioid Receptor in Complex with Gi Protein
Source: Int J Mol Sci. 2023 Aug 30;24(17):13430. doi: 10.3390/ijms241713430 (PMC10487971; doi:10.3390/ijms241713430)
Supplement: Supplementary file 1 [file ijms-24-13430-s001.zip › ijms-2510646-supplementary.pdf]

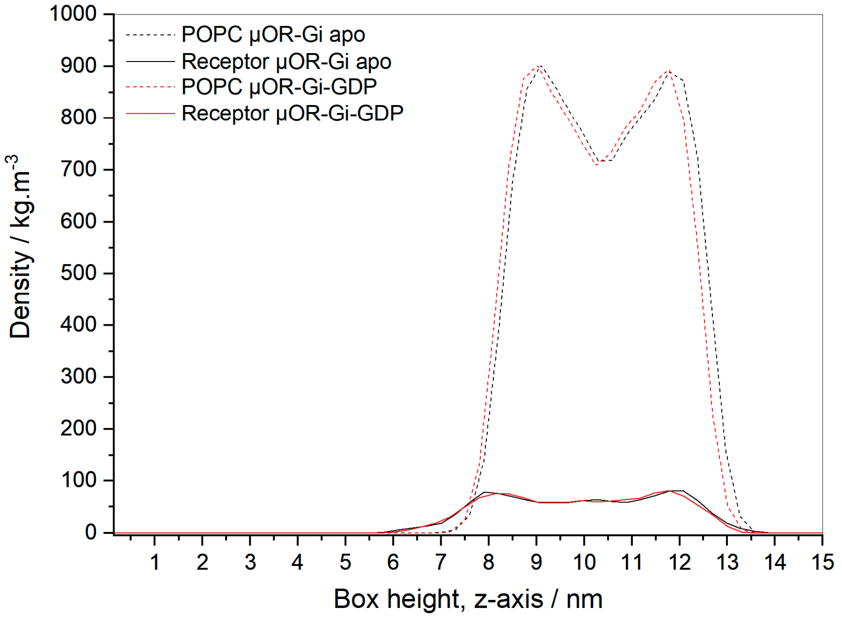

Figure S1 – Particle density along the simulation box z-axis. Receptor residues density (solid line) and POPC membrane (dashed line) are shown in black for theμOR-Gi apo system and in red for the μOR-Gi-GDP system.

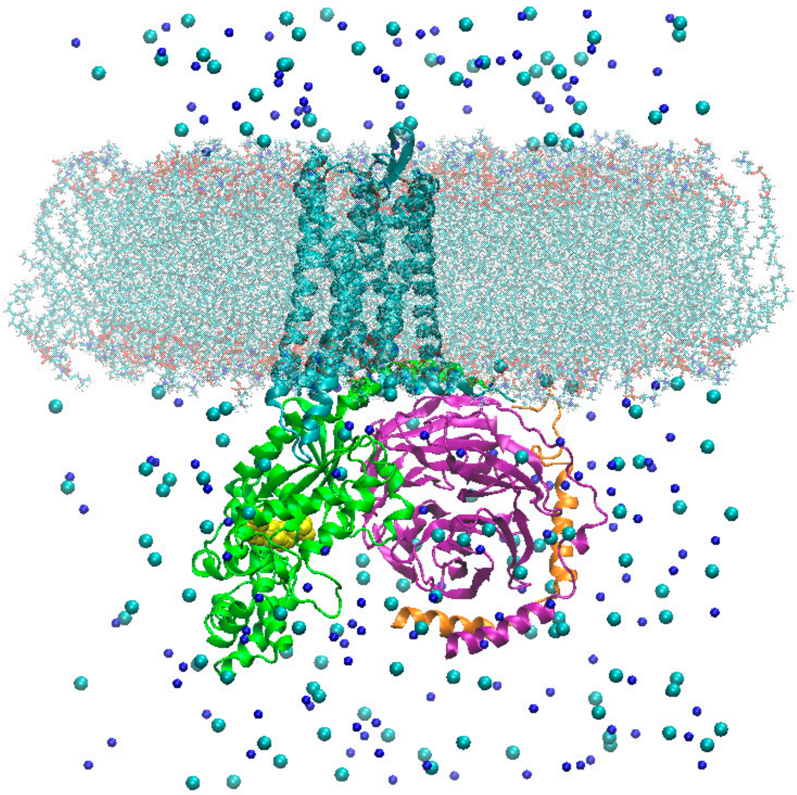

Figure S2 - Side view of the μOR-Gi-GDP system with GDP shown as a yellow surface, , Na^{+} ions (dark blue spheres), Cl^{-} ions (cyan spheres), and POPC represented as sticks. The proteins are shown as ribbons: μOR (in cyan), Gαi (in green), Gβ (in purple) and Gγ (in orange). The empty spaces above and below the membrane were filled with water molecules (not shown).

Table S1 – Distances between selected residues, related to Gαi helix 5 insertion assessment, from experimental μOR-Gi complex structures.

	Distances / Å	
**Structure (PDB id)**	**ASP147$^{3.32}$ Cα - Cα LEU348$^{G\alpha5}$**	**ARG165$^{3.50}$ Cα - Cα PHE354$^{G\alpha5}$**
6DDE	32.751	11.520
6DDF	32.973	11.474
7SBF	33.032	11.411
7SCG	34.460	12.204
7T2G	31.983	11.487
7T2H	32.755	11.692
7U2K	34.217	13.006
7U2L	32.787	11.513
8EF5	33.040	11.767
8EF6	33.242	11.805
8EFB	32.825	13.080
8EFL	33.146	13.270
8EFO	32.916	11.703
8EFQ	33.919	11.973
8F7Q	33.155	13.202
8F7R	32.886	11.869
Minimum value	31.983	11.411
Maximum value	34.460	13.270

Table S2 – Salt bridges found during Molecular Dynamics from μOR-Gi apo and μOR-Gi-GDP systems, exclusively.

μOR-Gi apo Salt Bridges	μOR-Gi-GDP Salt Bridges
K98$^{12.49}$-E349$^{8.56}$	R95$^{1.59}$-D312$^{G\alpha i}$
D272$^{6.27}$-R276$^{6.31}$	R182$^{4.40}$-E25$^{G\alpha i}$
R179$^{34.57}$-D350$^{G\alpha i}$	R185$^{4.43}$-E28$^{G\alpha i}$
R182$^{4.40}$-E38$^{G\alpha i}$	E270$^{6.25}$-R273$^{6.28}$
K269$^{6.24}$-E308$^{G\alpha i}$	E34$^{8.48}$-R345$^{8.52}$
R345$^{8.52}$-D312$^{G\beta}$	R345$^{8.52}$-D261$^{G\alpha i}$
D20$^{G\alpha}$-R24$^{G\alpha}$	D26$^{G\alpha i}$-K78$^{G\alpha i}$
E25$^{G\alpha}$-K67$^{G\alpha}$	E43$^{G\alpha i}$-R178$^{G\alpha i}$
E25$^{G\alpha}$-K70$^{G\alpha}$	E64$^{G\alpha i}$-K67$^{G\alpha i}$
D26$^{G\alpha}$-K29$^{G\alpha}$	R90$^{G\alpha i}$-E238$^{G\alpha i}$
E28$^{G\alpha}$-K67$^{G\alpha}$	R100$^{G\alpha i}$-D103$^{G\alpha i}$
K29$^{G\alpha}$-E33$^{G\alpha}$	D103$^{G\alpha i}$-R129$^{G\alpha i}$
R32$^{G\alpha i}$-E64$^{G\alpha i}$	K128$^{G\alpha i}$-D160$^{G\alpha i}$
R32$^{G\alpha i}$-D193$^{G\alpha i}$	R142$^{G\alpha i}$-E145$^{G\alpha i}$
K46$^{G\alpha i}$-D200$^{G\alpha i}$	D150$^{G\alpha i}$-R242$^{G\alpha i}$
E65$^{G\alpha i}$-K70$^{G\alpha i}$	D150$^{G\alpha i}$-K270$^{G\alpha i}$
E65$^{G\alpha i}$-K70$^{G\alpha i}$	D229$^{G\alpha i}$-R242$^{G\alpha i}$
D122$^{G\alpha i}$-R105$^{G\alpha i}$	D231$^{G\alpha i}$-K277$^{G\alpha i}$
D103$^{G\alpha i}$-K132$^{G\alpha i}$	E276$^{G\alpha i}$-K279$^{G\alpha i}$
R105$^{G\alpha i}$-E122$^{G\alpha i}$	E8$^{G\alpha i}$-R68$^{G\beta}$
E186$^{G\alpha i}$-K197$^{G\alpha i}$	D20$^{G\alpha i}$-K89$^{G\beta}$
D231$^{G\alpha i}$-R242$^{G\alpha i}$	D186$^{G\alpha i}$- K210$^{G\beta}$
D237$^{G\alpha i}$-R242$^{G\alpha i}$	E216$^{G\alpha i}$-K57$^{G\beta}$
K271$^{G\alpha i}$-E275$^{G\alpha i}$	R22$^{G\beta}$-E22$^{G\gamma}$
D315$^{G\alpha i}$-R313$^{G\alpha i}$	E17$^{G\gamma}$-K20$^{G\gamma}$
K29$^{G\alpha i}$-D76$^{G\beta}$	
K35$^{G\alpha}$-D76$^{G\beta}$	
K210$^{G\alpha i}$-D228$^{G\beta}$	
K257$^{G\alpha i}$-D246$^{G\beta}$	
R22$^{G\beta}$ -D258$^{G\beta}$	
R52$^{G\beta}$ -D333$^{G\beta}$	
D76$^{G\beta}$-K78$^{G\beta}$	
R96$^{G\beta}$-D118$^{G\beta}$	
D212$^{G\beta}$-R219$^{G\beta}$	
K280$^{G\beta}$-E47$^{G\gamma}$	

Table S3 – List of available Gi with GDP in Protein Data Bank

**Structure (PDB id)**	**year**	**resolution**	**organism (Gα)**
1GP2	1995	2.30 Å	*Rattus novergicus*
1GG2	1995	2.40 Å	*Rattus novergicus*
5TDH	2016	3.00 Å	*Homo sapiens*
6CRK	2018	2.00 Å	*Homo sapiens*

Table S4 – List of available μOR-Gi complexes in Protein Data Bank

Structure (PDB id)	year	resolution	organism (receptor)	organism (Gα)
6DDE	2018	3.50 Å	*Mus musculus*	*Homo sapiens*
6DDF	2018	3.50 Å	*Mus musculus*	*Homo sapiens*
7SBF	2022	2.90 Å	*Escherichia coli/ Mus musculus*	*Homo sapiens*
7SCG	2022	3.00 Å	*Mus musculus*	*Homo sapiens*
7T2G	2022	2.50 Å	*Escherichia coli/ Mus musculus*	*Homo sapiens*
7T2H	2022	3.20 Å	*Mus musculus*	*Homo sapiens*
7U2K	2023	3.30 Å	*Mus musculus*	*Homo sapiens*
7U2L	2023	3.20 Å	*Mus musculus*	*Homo sapiens*
8EF5	2022	3.30 Å	*Homo sapiens*	*Homo sapiens*
8EF6	2022	3.20 Å	*Homo sapiens*	*Homo sapiens*
8EFB	2022	3.20 Å	*Homo sapiens*	*Homo sapiens*
8EFL	2022	3.20 Å	*Homo sapiens*	*Homo sapiens*
8EFO	2022	2.80 Å	*Homo sapiens*	*Homo sapiens*
8EFQ	2022	3.30 Å	*Homo sapiens*	*Homo sapiens*
8F7Q	2023	3.22 Å	*Homo sapiens*	*Homo sapiens*
8F7R	2023	3.28 Å	*Homo sapiens*	*Homo sapiens*

Table S5 – Number of particles constituting the model systems used in the simulations.

Model	Receptor residues	Gαi residues	Gβγ dimer residues	POPC molecules	Na^+ íons	Cl^- íons	Water molecules	Total atoms
**μOR-Gi apo**	306	350	440	442	138	138	50708	228619
**μOR-Gi-GDP**	306	350	440	442	138	135	53990	238526

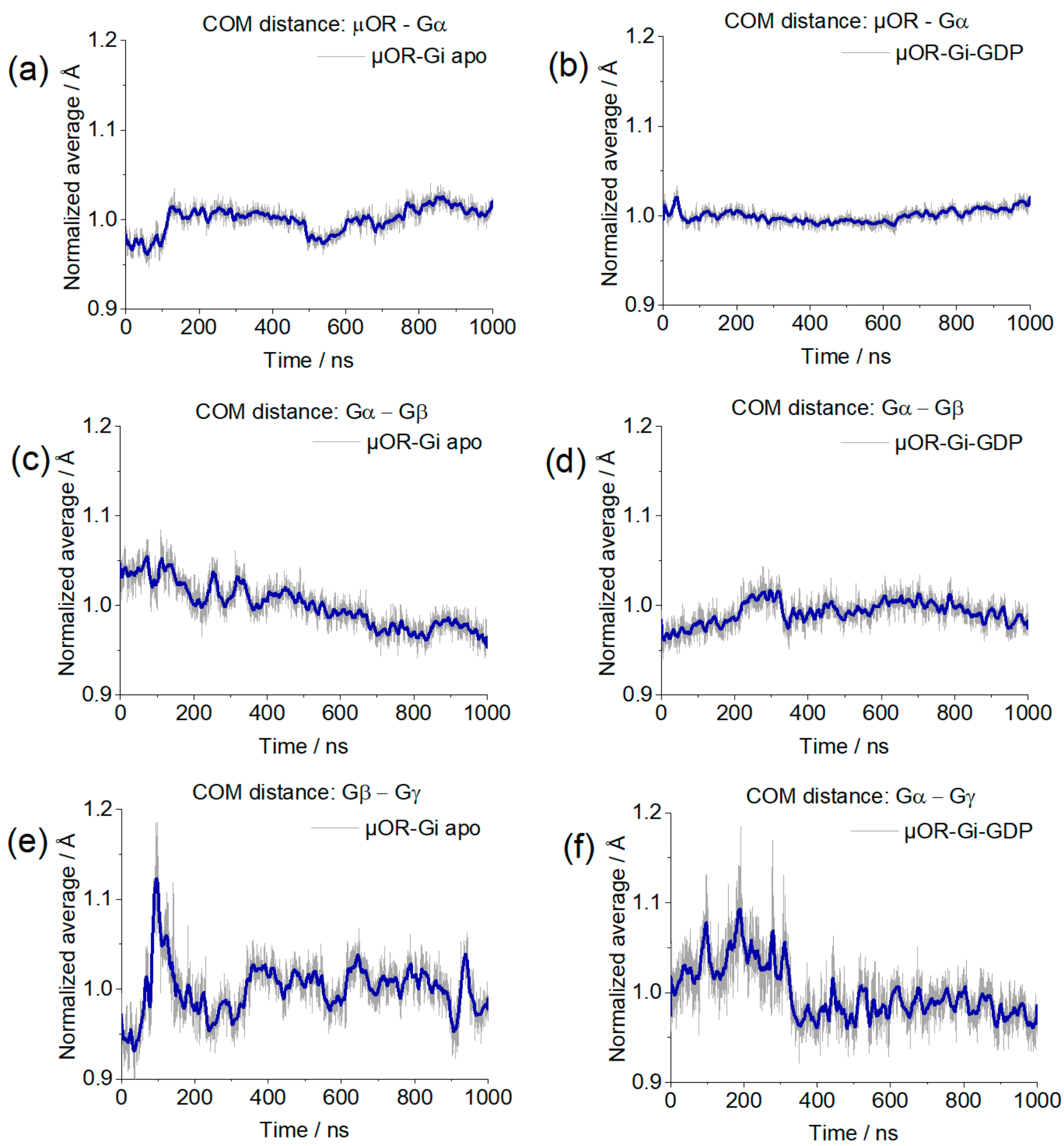

Figure S3 – Normalized distances between COM for selected portions/systems: a) μOR and Gαi from μOR -Gi apo; b) μOR and Gαi from μOR -Gi-GDP; c) Gαi and Gβ from μOR -Gi apo; d) Gαi and Gβ from μOR-Gi-GDP; e) Gβ and Gγ from μOR -Gi apo; f) Gβ and Gγ from μOR-Gi-GDP.

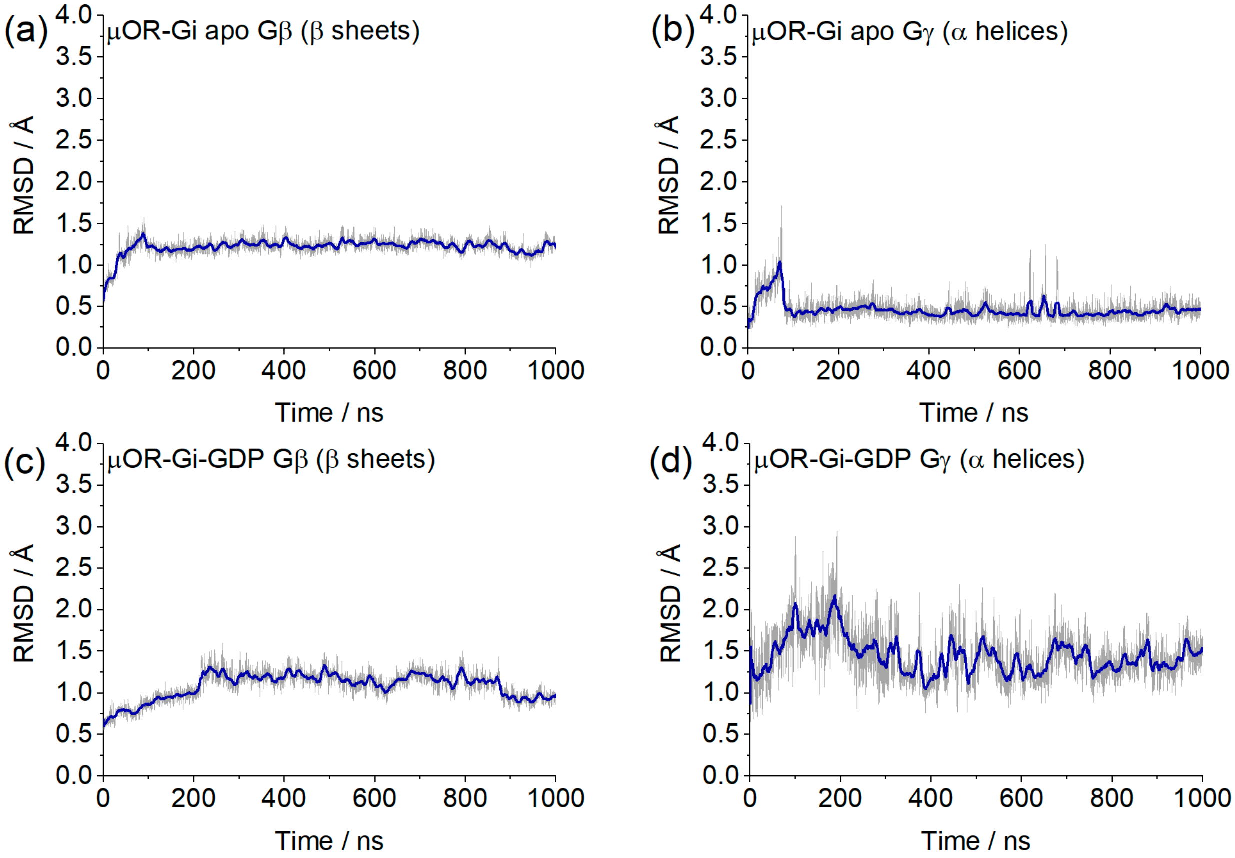

Figure S4 - RMSD values from Cα of the Gβ and Gγ protein for selected portions/systems: a) β-sheets Gβ/μOR -Gi apo; b) β-sheets Gβ/μOR-Gi GDP; c) α-helices Gγ/μOR-Gi apo and d) α-helices Gγ/μOR-Gi-GDP
